# Rapid coronary artery stent computational simulation using the simplex deformable model

**DOI:** 10.1088/1873-4030/ae6a9e

**Published:** 2026-05-29

**Authors:** Changkye Lee, Shijia Zhao, Wei Wu, Rakshita Ramesh Bhat, Priyansh Patel, Yash Vardhan Trivedi, Parth Munjal, Yoshinobu Murasato, Yiannis S Chatzizisis

**Affiliations:** 1 Center for Digital Cardiovascular Innovations, Division of Cardiovascular Medicine, University of Miami, Miami, FL, United States of America; 2 Department of Cardiology, National Hospital Organization Kyushu Medical Center, Fukuoka, Japan

**Keywords:** simplex deformable model, rapid simulation, patient-specific coronary artery, finite element analysis

## Abstract

The objective of this study was to present and demonstrate the feasibility of a simplex deformable model (SDM) for patient-specific coronary stent simulations in two patient-specific cases. Patient-specific 3D artery anatomies were converted into triangular meshes and then transformed into 2-simplex meshes. For each vertex, three neighboring vertices were used to compute outward and inward normal vectors to model stent expansion and arterial wall resistance. Internal forces representing stent mechanics were computed during expansion, and external forces incorporating non-linear hyperelastic arterial behavior were applied after stent-wall contact. Two patient-specific arteries were simulated using the SDM framework, with the finite element method (FEM) as the reference. Relative to FEM, the SDM achieved 360–670 times faster computational time, and reduced vertex counts by 9.5–12.6 times. Bland–Altman analysis showed small mean biases in mean lumen diameter ($-$0.029 mm for Artery 1; $-$0.043 mm for Artery 2), and root mean square error was small (0.063 mm for Artery 1; 0.098 mm for Artery 2), and the mean absolute relative error was also low (1.75% for Artery 1; 2.31% for Artery 2). In conclusion, this proof-of-concept study in two patient-specific 3D coronary anatomies suggests that the proposed SDM-based simulations can achieve overall agreement with FEM, while substantially reducing computational time and mesh size.

## 1. Introduction

Percutaneous coronary intervention is a widely used minimally invasive approach to effectively treating obstructive coronary artery disease (Abubakar *et al* 2023, Samant *et al* 2023). The procedure typically begins with insertion of a catheter carrying a stent pre-mounted on an inflatable balloon; balloon inflation expands the arterial wall and enlarges the lumen to restore blood flow. After inflation, the balloon is deflated and removed, while the stent helps maintain the expanded lumen and prevents elastic recoil of the artery. Clinical outcomes after stent implantation depend on multiple factors, including stent design and radial strength, deployment accuracy, and the risk of in-stent restenosis (Karanasiou *et al* 2014, Wu *et al* 2022, Kapoor *et al* 2024).

Computational simulations provide a valuable means to study stent deployment by predicting stent-artery mechanical interaction and enabling evaluation of outcomes such as malapposition and restenosis-related indicators (Martin and Boyle 2011, 2013). They also support systematic exploration of device- and procedure-related variables, including stent designs, material properties, and flexibility (Chiastra *et al* 2016, He *et al* 2020, Hoddy *et al* 2022). For patient-specific prediction, accurate anatomical geometry and realistic mechanical behavior are essential (Samant *et al* 2021, Zhao *et al* 2021). The finite element method (FEM) has therefore been widely used for deployment simulations, particularly for non-linear large-deformation analyses (Auricchio *et al* 2001, Zahedmanesh *et al* 2010). To reduce computational expense, simplified balloon representations and reduced-dimensional stent models have been proposed (Geith *et al* 2019, Krewcun *et al* 2019). Nevertheless, FEM-based deployment simulations often remain resource-intensive for patient-specific, high-resolution analyses, especially when fine discretization is required to resolve contact and non-linear material response (Datz *et al* 2025, Wu *et al* 2025).

The simplex deformable model (SDM), originally developed for medical image reconstruction (Delingette 1994, Montagnat and Delingette 2005), offers an efficient representation of deforming meshes and has therefore attracted interest for virtual stenting (Flórez-Valencia *et al* 2004, Larrabide *et al* 2008, Paliwal *et al* 2016). Existing SDM-based stenting frameworks, however, face an enduring trade-off between mechanical accuracy and computational efficiency. On one hand, fast SDM formulations often use simplified or data-calibrated mechanical components; for example, Chen *et al* (2018) proposed a TEVAR framework based on a data-fitted radial force–displacement relationship, which may limit predictive accuracy for stress-driven deformation and recoil when device or material properties vary. On the other hand, incorporating FEM-like force evaluations can improve mechanical behavior, but increases computational cost, potentially reducing the efficiency advantage of the SDM (Djukic *et al* 2019). To address this trade-off, the present study advances the SDM for balloon-expandable coronary stents by embedding stent material and structural parameters directly into the internal force term, enabling mechanics-informed deployment prediction in patient-specific coronary anatomy without resorting to full FEM-based force evaluation.

Motivated by the need for patient-specific deployment tools that are both mechanically credible and computationally efficient, the aim of this study was to present and validate a mechanics-informed SDM-based workflow that achieves FEM-comparable accuracy, while retaining the computational efficiency of simplex-based deformation. We benchmarked the proposed SDM against FEM on patient-specific coronary arteries by comparing arterial displacement fields and computational time.

## 2. Methods

All methods were carried out in accordance with the relevant guidelines and regulations. The use of these geometries was approved by the ethics committee of Teikyo University (IRB approval number #15-159-2). Informed consent was obtained from all participants.

The basic idea of the proposed SDM for simulating balloon-expandable stent deployment in patient-specific coronary arteries using a 2-SDM can be summarized as follows. First, patient-specific 3D arterial geometries were reconstructed, followed by surface mesh extraction and conversion to 2-simplex representations (polygonal meshes with three neighbors per vertex), enabling efficient deformation while maintaining geometric consistency. The stent mesh was then generated by inward projection along the artery centerline. Stent expansion was modeled by an internal force that drives uniform radial deployment outward. In contrast, wall resistance was introduced as an inward external force after stent-lumen wall contact was detected. Of note, the internal force incorporates the mechanical properties of the stent, and the external force accounts for non-linear hyperelastic large deformations. To address the feasibility of the SDM in terms of accuracy and efficiency, predictions were compared against FEM simulations in Abaqus, used as a reference solution. Two clinical coronary geometries (LAD and left circumflex (LCX)) were analyzed, and the SDM was intentionally run with mesh densities different from the finer Abaqus meshes to assess robustness. To quantitatively compare the mean lumen diameter (MLD) predicted by the SDM with that from FEM, frame-wise MLD values were evaluated along the full length of each patient-specific artery. Agreement across the artery length was assessed using Bland–Altman analysis and three numerical error metrics: maximum absolute error, root mean square error (RMSE), and mean absolute relative error (MARE). In addition, five representative cross-sections were selected for localized comparison between the SDM and FEM.

### 2.1. Simplex deformable mesh

**Figure 1.**
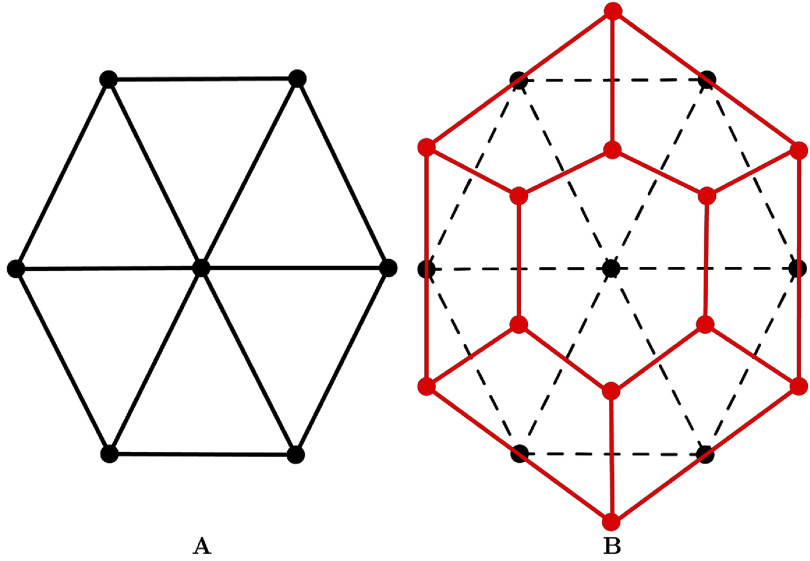
Construction of the 2-simplex meshes. (A) Triangular meshes; and (B) 2-simplex meshes. Black lines and circles represent the initial triangular mesh, and red lines and circles represent the discretized polygonal mesh.

The SDM was employed to represent the surface geometry, which allows the mean curvature and the outward normal vector to be evaluated at each vertex. Unlike element-based parametric formulations, SDM is not based on finite elements; therefore, it does not require stiffness-matrix assembly or repeated isoparametric mapping updates during mesh deformation. This property reduces computational cost and generally yields faster simulations than element-based numerical approaches. A key property of 2-simplex meshes is that their connectivity is dual to a triangulation (Delingette 1999) (figure 1).

For a $k$-simplex mesh embedded in $\mathbb{R}^d$, the parameter $k$ determines the vertex connectivity such that each vertex has exactly $(k+1)$ neighboring vertices, while $d$ denotes the dimension of the embedding space. In this study, we use $k = 2$ and $d = 3$: the surface was represented by a 2-simplex mesh embedded in three-dimensional Euclidean space ($\mathbb{R}^3$), where each vertex had exactly three neighbors. Accordingly, as illustrated in figure 1, an initial triangular surface mesh was converted into a polygonal (2-simplex) mesh in which each vertex $P_i$ is connected to precisely three neighboring vertices $\left(P_{N_1(i)},\,P_{N_2(i)},\,P_{N_3(i)}\right)$. These three neighbors define a local tangent plane at vertex $P_i$, from which the unit normal vector $\mathbf{n}_i$ is computed as:

\begin{equation*} \mathbf{n}_i = \dfrac{P_{N_1\left(i\right)}\wedge P_{N_2\left(i\right)} + P_{N_2\left(i\right)}\wedge P_{N_3\left(i\right)} + P_{N_3\left(i\right)}\wedge P_{N_1\left(i\right)}}{\left\|P_{N_1\left(i\right)}\wedge P_{N_2\left(i\right)} + P_{N_2\left(i\right)}\wedge P_{N_3\left(i\right)} + P_{N_3\left(i\right)}\wedge P_{N_1\left(i\right)}\right\|}.\end{equation*}

**Figure 2.**
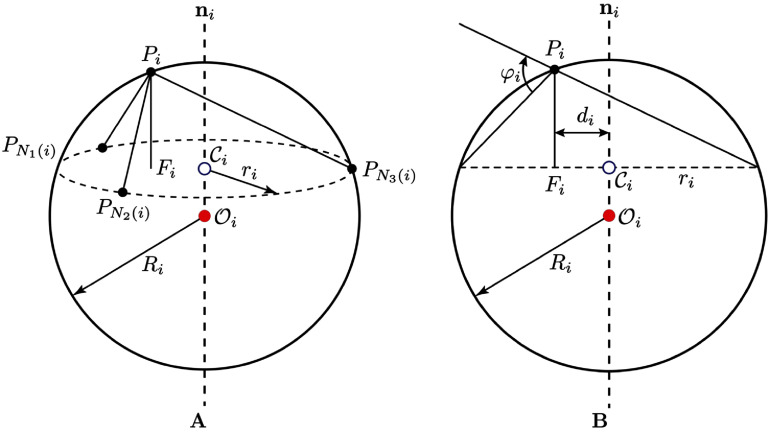
Geometric definition of the 2-simplex mesh. (A) The circumscribed sphere with radius $R_i$ and centroid $\mathcal{O}_i$, together with the circumscribed circle with radius $r_i$ and centroid $\mathcal{C}_i$; and (B) the planar projection, where $F_i$ is the projection of vertex $P_i$ onto the plane and $\varphi_i$ is the simplex angle.

As shown in figure 2, the neighboring vertices $\left(P_{N_1(i)},\,P_{N_2(i)},\,P_{N_3(i)}\right)$ form a circumscribed circle with radius $r_i$ and centroid $\mathcal{C}_i$. In addition, the four vertices $\left(P_i,\,P_{N_1(i)},\,P_{N_2(i)},\,P_{N_3(i)}\right)$ define a circumscribed sphere with radius $R_i$ and centroid $\mathcal{O}_i$. The simplex angle $\varphi_i = \angle\left(P_i,\,P_{N_1(i)},\,P_{N_2(i)},\,P_{N_3(i)}\right)$ is then defined by:

\begin{equation*} \begin{split} \sin\left(\varphi_i\right) &amp; = \dfrac{r_i}{R_i}\, \mathrm{sign}\left(\left(P_{N_1\left(i\right)}-P_i\right)\cdot\mathbf{n}_i\right)\\ \cos\left(\varphi_i\right) &amp; = \dfrac{\left\|\mathcal{C}_i-\mathcal{O}_i\right\|}{R_i}\,\mathrm{sign}\left(\left(\mathcal{C}_i-\mathcal{O}_i\right)\cdot\mathbf{n}_i\right) \end{split}\end{equation*}

where the simplex angle ranges as $\varphi_i\in\left[-\pi,\,\pi\right]$.

In the SDM, vertices are treated as lumped physical entities that evolve according to Newton’s second law under the action of model-defined driving terms. Importantly, the ‘forces’ in SDM are not the internal residual forces or applied external loads of classical FEM, which are obtained via stress integration and stiffness-matrix assembly. Instead, in the SDM framework, they are algorithmic, force-like terms constructed from local geometric measures (e.g. normals and curvature) and interaction laws (e.g. contact with the arterial wall) to control mesh deformation. The vertex dynamics are written as:

\begin{equation*} m\dfrac{\partial^2 P_i}{\partial t^2} = -\gamma\dfrac{\partial P_i}{\partial t} + \mathbf{F}_i^{\mathrm{int}} + \mathbf{F}_i^\mathrm{ext},\end{equation*}

where $m$ is the lumped mass associated with vertex $P_i$ ($P_i\in\mathcal{P}$), $\gamma$ is a numerical damping coefficient, $t$ is time, and $\mathbf{F}_i^{\mathrm{int}}$ and $\mathbf{F}_i^\mathrm{ext}$ denote the internal (model/regularization) and external (interaction/loading) SDM force (driving) terms, respectively. To solve a numerical approximation of equation (3), the SDM utilizes the centered difference method, as given by:

\begin{equation*} P_i^{t+1} = P_i^t + \left(1-\gamma\right)\left(P_i^t - P_i^{t-1}\right) + \alpha \mathbf{F}_i^\mathrm{int} + \beta \mathbf{F}_i^\mathrm{ext}\end{equation*}

where $\alpha$ and $\beta$ are the force weight coefficients, and both internal and external forces are calculated at each time step $t$. Note that the dimensions of the displacements are included in both the internal and external forces in equation (4). The details of the discretization schemes and relevant equations can be found in appendix A and in Delingette (1999), Montagnat *et al* (2000).

To model balloon-expandable deployment efficiently within the present SDM framework, we adopted the following assumptions: (a) the stent exhibits purely elastic behavior; (b) recoil after full expansion and balloon deflation is neglected; and (c) the balloon and stent expand simultaneously and uniformly. Under these assumptions, the internal driving term represents an outward expansion tendency of the stent, whereas the external driving term is activated only after stent-artery contact and represents the inward resistance of the arterial wall. Assuming elastic behavior, the internal driving term was defined through a relationship between radial force and radial displacement as:

\begin{equation*} \mathbf{F}_i^{\mathrm{int}} = \dfrac{k_\mathrm{Stn}}{F_\mathrm{RF}D_\mathrm{Stn}} \left(\left(1-\chi\right)D_\mathrm{Stn}-\left|\delta P_i\right|\right)\mathbf{n}_i\end{equation*}

where $k_\mathrm{Stn}$ denotes the radial rigidity of the stent, $F_\mathrm{RF}$ is the radial force exerted by the stent, $D_\mathrm{Stn}$ is the target diameter of the fully expanded stent, $\delta P_i$ is the radial distance between the stent centerline and vertex $P_i$, $\chi$ is a dimensionless scaling factor, and $\mathbf{n}_i$ is the outward unit normal given in equation (1). Representing the stent’s radial rigidity, $k_\mathrm{Stn}$, was calculated as:

\begin{equation*} k_\mathrm{Stn} = \dfrac{3E_\mathrm{Stn}I}{L^3}\end{equation*}

where $E_\mathrm{Stn}$ is the Young’s modulus of the stent, $L$ is the stent’s length, and $I = \pi D_\mathrm{Stn}^4 / 64$ is the moment of inertia. The radial force $F_\mathrm{RF}$ is given by $F_\mathrm{RF} = F_\mathrm{unit}\times L$, where $F_\mathrm{unit}$ is the force per unit length calculated as $F_\mathrm{unit} = P_\mathrm{nom}\times D_\mathrm{Stn}$. Note that $D_\mathrm{Stn}$ is the expected fully expanded diameter of the stent. The coefficient $\chi$ in equation (5) is determined by $\chi = T \times \delta$, where $T$ is a threshold value indicating the percentage expansion of the artery’s diameter, and $\delta$ is the normalized inflection position. This normalized inflection position was computed as:

\begin{equation*} \delta = \dfrac{L}{2\pi t k_\mathrm{Stn}r_\mathrm{Stn}}\end{equation*}

where $t$ is the thickness of stent’s strut. Note that this study established an SDM-aided virtual stent simulation paradigm using updated SDM motion equations and a contact-activated external resistance force. The key contribution of the proposed framework lies in the internal force term in equations (5)–(7). In contrast to data calibrated radial force–displacement formulations, we computed the stent’s radial rigidity from the material modulus and stent properties (e.g. $E$, $I$, strut thickness, etc), yielding a mechanics-informed internal term tailored to balloon-expandable coronary stents and allowing parameters to be assigned directly from device specifications. As a result, stents with different geometric (length, diameter, strut thickness) and material (Young’s modulus, Poisson’s ratio) characteristics can be straightforwardly incorporated into the SDM framework.

Contact was assumed once the distance between a stent vertex and the arterial wall became smaller than the stent-strut diameter. After contact, the arterial wall resisted further expansion, and the external driving term was computed as:

\begin{equation*} \mathbf{F}_i^\mathrm{ext} = \dfrac{\nu\sigma_{\mathrm{Vsl}}}{2\sigma_{\mathrm{res}}} \left| \left(P_{N_2\left(i\right)}-P_{N_1\left(i\right)}\right)\times\left(P_{N_3\left(i\right)}-P_{N_1\left(i\right)}\right) \right|\mathbf{n}_i\end{equation*}

where $\nu$ is Poisson’s ratio, $\sigma_\mathrm{Vsl}$ is the arterial stress, and $\sigma_\mathrm{res}$ is the arterial resistance stress. The resistant stress $\sigma_\mathrm{res}$ in equation (8) was assumed a circumferential ratio of $\lambda = 1.4$, which corresponds to a 40% expansion of the artery’s original diameter for example. Of note, in the pre-contact phase, the stent expands without interaction with the artery wall; therefore, $\mathbf{F}^\mathrm{ext} = 0$.

### 2.2. Numerical implementation

In the present study, the stent was modeled as purely elastic; therefore, its elastic-plastic behavior and the resulting recoil were not considered. The stent material was defined as a cobalt alloy with radius $r_\mathrm{Stn} = 0.5$ mm, elastic modulus $E_\mathrm{Stn} = 233$ GPa, and Poisson’s ratio $\nu = 0.35$, and a nominal balloon pressure of $P_\mathrm{nom} = 9$ atm was used. To capture the mechanical behavior of the artery, a reduced polynomial hyperelastic model (appendix B) was used, with coefficients listed in table 1 (Zhao *et al* 2021).

**Table 1.** Coefficients for the artery wall material.

Type	$C_{10}$ (MPa)	$C_{20}$ (MPa)	$C_{30}$ (MPa)	$C_{40}$ (MPa)	$C_{50}$ (MPa)	$C_{60}$ (MPa)
Normal wall	$6.52\times10^{-3}$	$4.89\times10^{-2}$	$9.26\times10^{-3}$	$7.60\times10^{-1}$	$-4.30\times10^{-1}$	$8.69\times10^{-2}$

**Figure 3.**
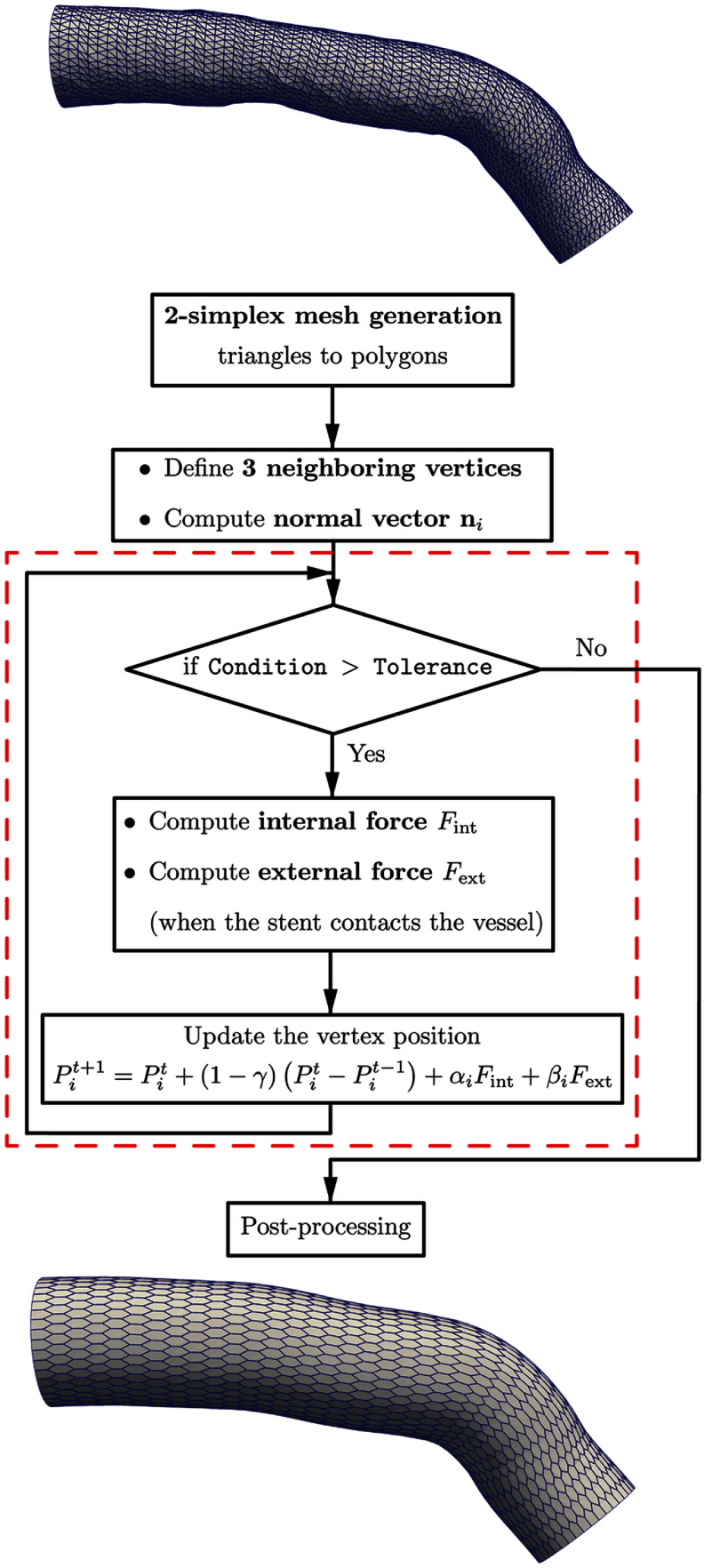
Schematic flowchart of the proposed model.

The overall workflow of the proposed SDM-based stent deployment simulation is summarized in figure 3. First, patient-specific 3D arterial geometries were reconstructed by fusing angiography and optical coherence tomography images. Tetrahedral volume meshes were then generated using the ANSYS ICEM mesh generator. During SDM pre-processing, triangular surface meshes were extracted from the tetrahedral artery model and converted into polygonal meshes to obtain 2-simplex representations. The 2-simplex mesh of the stent was generated by projecting the polygonal artery mesh inward along the arterial centerline according to the stent diameter. For both the stent and artery meshes, each vertex was assigned three neighboring vertices, and the corresponding inward/outward normal vectors $\mathbf{n}_i$ were computed.

During the main simulation, the internal driving term $F_\mathrm{int}$ was computed under the assumption of uniform stent expansion. Once the expanding stent contacted the arterial wall, the external driving term $F_\mathrm{ext}$ was activated to represent the inward mechanical resistance of the artery. At each time step, vertex positions $P_i$ were updated using equation (4). The proposed SDM was implemented in C++ and executed on an Ubuntu workstation equipped with an Intel® Core$^\mathrm{TM}$ i9-10 900X processor (3.70 GHz) and 32 GB of memory.

To evaluate the proposed SDM, a FEM reference solution was obtained using Abaqus (Dassault Systèmes Simulia Corp., Rhode Island, United States) (Dassault Systèmes Simulia Corp. 2024), which has demonstrated high accuracy in vascular intervention simulations (Poletti *et al* 2022, Antonini *et al* 2025). The Abaqus model used in this study was previously verified against 10 clinical cases (Zhao *et al* 2021). In Abaqus, the artery was modeled using four-node tetrahedral solid elements (C3D4), while the stent and balloon were represented using solid and membrane elements, respectively.

## 3. Results

Two patient-specific arterial geometries were analyzed: (a) a diseased left anterior descending (LAD) artery (Artery 1) and (b) a diseased LCX artery (Artery 2). For verification, arteries 1 and 2 were simulated using SDM meshes with lower density than the finer meshes used in the corresponding Abaqus models. This design was intended to assess the accuracy and robustness of the SDM against high-resolution FEM reference solutions.

### 3.1. Patient-specific Artery 1

**Figure 4.**
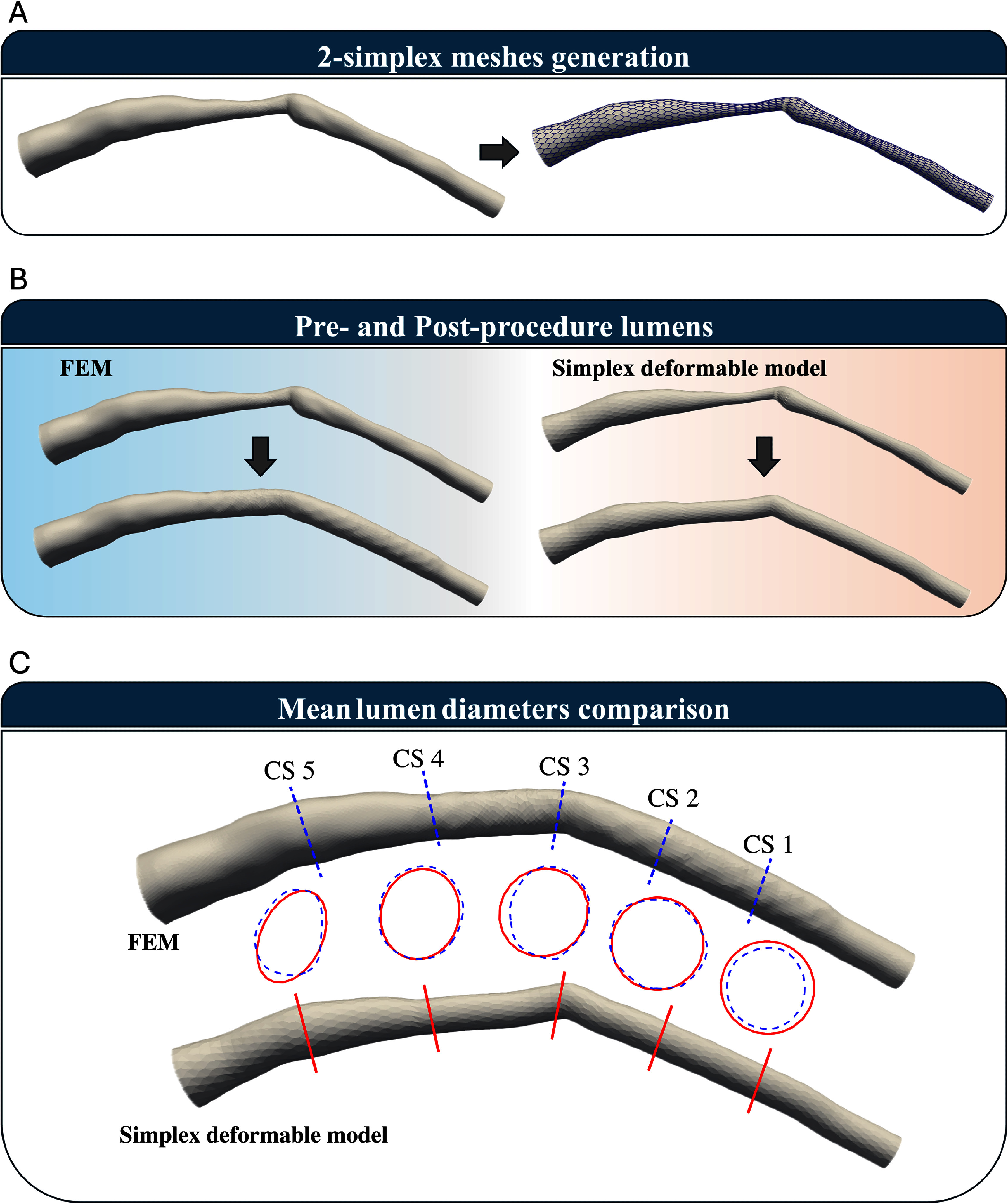
2-simplex meshing and cross-section for Artery 1. (a) Conversion of the 3D artery into polygonal meshes for applying the 2-simplex meshes to Artery 1; (b) comparison of lumen shapes pre- and post-procedure; and (c) measured artery diameters at five designated cross-sections (CS1–CS5).

The first patient-specific 3D artery (Artery 1) and its discretized 2-simplex meshes are shown in figure 4(a). The 2-simplex mesh of the stent was initially generated based on centerline vertices and the stent’s radius in its crimped state. The stent vertices were then projected onto the artery surface, and the nearest existing artery vertices were identified to construct the 2-simplex mesh of the artery.

**Figure 5.**
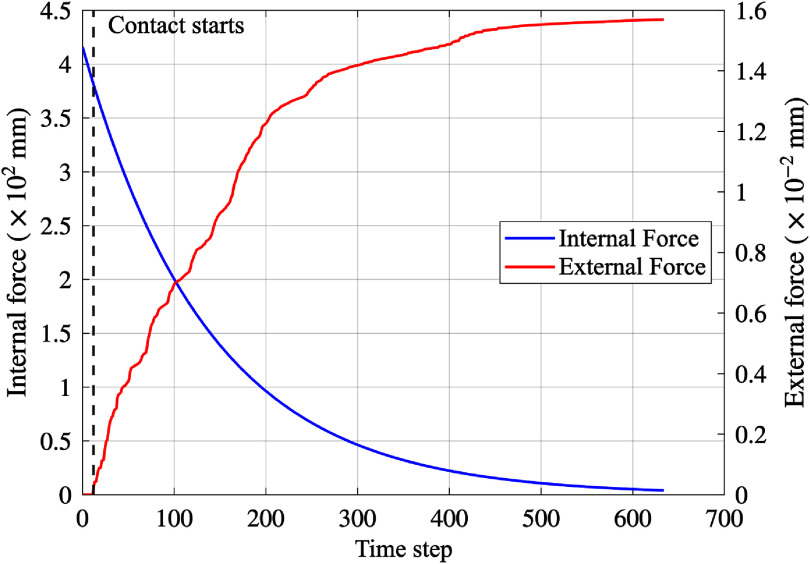
Time-step history of the total internal and external force magnitudes (sum of nodal magnitudes) for Artery 1. The internal force is shown in red, the external force is shown in blue, and the black dashed line indicates the onset of contact.

In this analysis, a 34.0 mm-long stent was modeled to expand to a diameter of 2.15 mm in both the SDM and FEM simulations. Figure 4(b) shows the lumen shapes before and after the procedure, as obtained by the SDM and FEM. Figure 4(c) presents artery diameters measured at five designated cross-sections (CS1–CS5), facilitating a direct comparison between the two methods. Despite having significantly fewer vertices, the SDM results closely matched those from FEM. Figure 5 shows the time-step history of the internal and external forces (reported as the sum of nodal magnitudes) during the expansion process. As shown in figure 5, the external force is zero before contact; once the stent contacts the arterial wall, the external force increases. Meanwhile, the internal force steadily decreases throughout the entire process, whereas the external force increases after contact, reflecting the growing stent-artery interaction during expansion. The SDM used only 3080 vertices and completed the simulation in approximately 2 s, whereas FEM, with 29 210 vertices, required about 1.8 h. Simulation times for both methods are summarized in table 2.

**Table 2.** Comparison of simulation times of Artery 1 between the SDM and FEM.

Method	Vertex count	Process time (s)			Total time (s)
		Pre-process	Main process	Post-process	
SDM	3080	2.93	2.03	17.02	21.98
FEM	29 210	N/A	6679.10		6679.10

N/A: Mesh generation time in FEM (Abaqus) is excluded, focusing solely on the solver time for the expansion phase.

### 3.2. Patient-specific Artery 2

**Figure 6.**
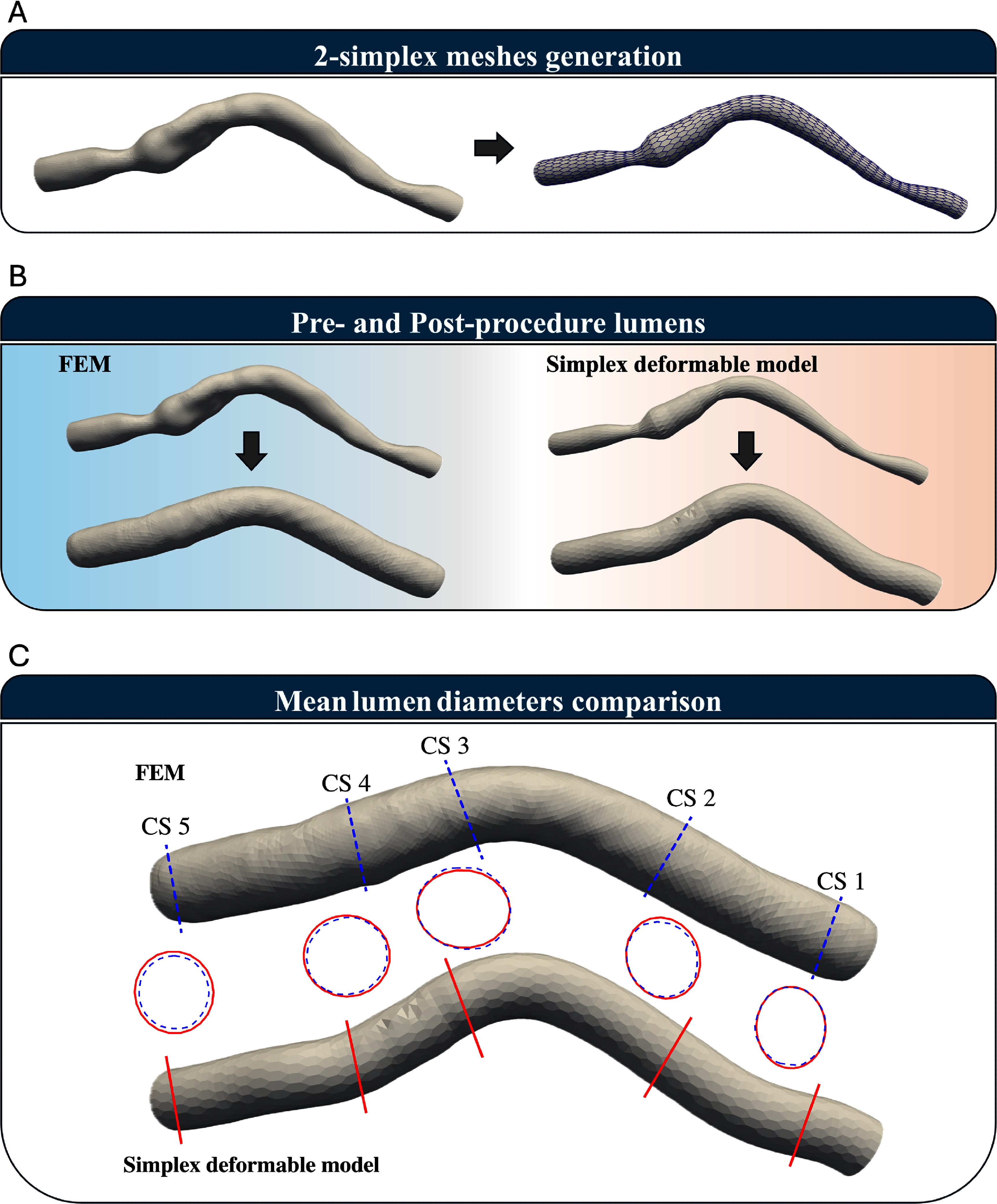
2-simplex meshing and cross-section for Artery 2. (a) Conversion of the 3D artery into polygonal meshes for applying the 2-simplex meshes to Artery 2; (b) comparison of lumen shapes pre- and post-procedure; and (c) measured artery diameters at five designated cross-sections (CS1–CS5).

**Figure 7.**
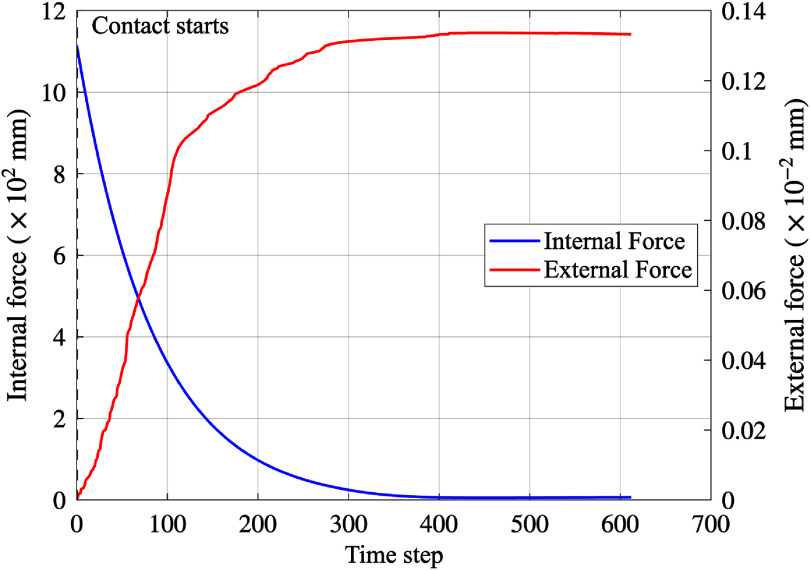
Time-step history of the total internal and external force magnitudes (sum of nodal magnitudes) for Artery 2. The internal force is shown in red, the external force is shown in blue, and the black dashed line indicates the onset of contact.

Figure 6(a) illustrates the initial arterial geometry of Artery 2 using 2-simplex meshes. As with Artery 1, the SDM generated these meshes from initial triangular meshes aligned with the arterial centerline. For comparison, the Abaqus simulation was conducted using C3D4 elements. The SDM, based on a 2-simplex mesh with 2244 vertices, was compared against the FEM simulation using a 3D patient-specific artery with 28 232 vertices. Both methods modeled the patient’s artery with a stent of 3.0 mm nominal diameter and 34.0 mm length, subjected to an internal pressure of 9 atm, targeting an expansion diameter of 2.90 mm. Figure 6(b) compares the lumen geometries of Artery 2 before and after the intervention, as obtained by the SDM and FEM. Figure 6(c) compares artery diameters measured at five designated cross-sections to validate the SDM against FEM. The results from the SDM are evaluated alongside those from FEM simulations using solid element. The force history for Artery 2 is provided in figure 7, where forces are reported as the sum of nodal magnitudes. Compared with Artery 1, Artery 2 begins with a preloaded stent-artery interaction due to the narrowed lumen contacting the deflated stent. Consequently, the external force is non-zero at initiation and grows during expansion, while the internal force drops as the stent configuration stabilizes.

Table 3 summarizes the computational costs of the SDM at each stage in comparison to the FEM simulations. Similar to the Artery 1, the proposed SDM completed the entire process-from mesh generation to post-processed image output-in approximately 10 s, whereas the FEM simulation required about 1.86 h.

**Table 3.** Comparison of simulation times of Artery 2 between the SDM and FEM.

Method	Vertex count	Process time (s)			Total time (s)
		Pre-process	Main process	Post-process	
SDM	2244	2.06	1.03	6.87	9.96
FEM	28 232	N/A	6684.0		6684.0

N/A: Mesh generation time in FEM (Abaqus) is excluded, focusing solely on the solver time for the expansion phase.

### 3.3. Agreement between the SDM and FEM

**Figure 8.**
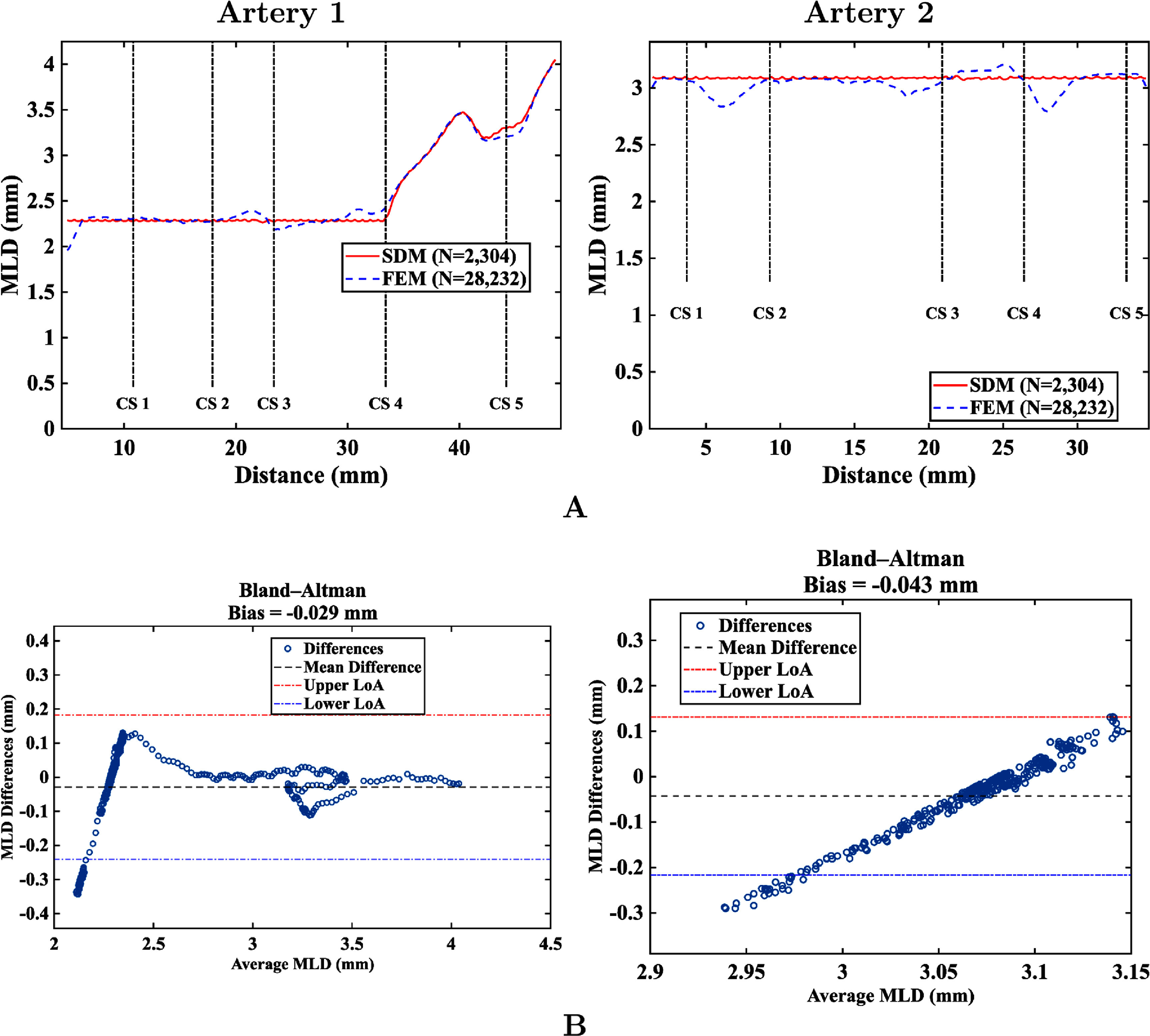
Statistical comparison results for Artery 1 and Artery 2. (a) Mean lumen diameter (MLD); (b) Bland–Altman plot.

Figure 8(a) illustrates the MLDs for Artery 1 and Artery 2, comparing the results from the SDM and FEM simulations. Figure 8(b) presents the Bland–Altman plot to visualize the agreement in MLDs, together with the mean bias and the 95% limits of agreement (LoA). In figure 8(b), the horizontal axis shows the average of the two methods (the SDM and FEM), and the vertical axis shows their difference, $\Delta = \mathrm{SDM}-\mathrm{FEM}$. The black dashed horizontal line indicates the mean difference (bias), while the red and blue dashed lines indicate the 95% LoA, which provide the typical range in which most $\Delta$ values (i.e. SDM minus FEM) are expected to fall. Together with the small bias, the Bland–Altman results suggest overall agreement between the SDM and FEM.

The measured MLDs at the five designated cross-sections, summarized in tables 4 and 5, provide representative sectional comparisons between the SDM and FEM simulations. These tables, together with figure 8(a), further show that local discrepancies were present in both artery models. These discrepancies arose from differences in the expansion mechanisms between the SDM and FEM because the SDM applies uniform expansion simultaneously along the model. Table 6 summarizes the frame-wise prediction errors of the SDM relative to FEM in terms of maximum absolute error, RMSE, and MARE. For both artery anatomies, the maximum absolute error was approximately 0.3 mm and the MARE ranged from 1.75% to 2.31%. The RMSE values were below 0.1 mm, indicating small overall differences relative to FEM.

**Table 4.** Measured mean lumen diameters at five designated cross-sections for Artery 1.

Method	CS 1 (mm)	CS 2 (mm)	CS 3 (mm)	CS 4 (mm)	CS 5 (mm)
SDM	3.319	2.323	2.306	2.306	2.302
FEM	3.311	2.441	2.251	2.304	1.973

**Table 5.** Measured mean lumen diameters at five designated cross-sections for Artery 2.

Method	CS 1 (mm)	CS 2 (mm)	CS 3 (mm)	CS 4 (mm)	CS 5 (mm)
SDM	3.110	3.110	3.114	3.111	3.101
FEM	3.049	2.936	3.099	2.883	2.784

**Table 6.** Frame-wise prediction error estimation for mean lumen diameter in Artery 1 and Artery 2.

Method	$\mathrm{max}|e|$ (mm)	RMSE (mm)	MARE (%)
SDM	0.332	0.063	1.75
FEM	0.289	0.098	2.31

$\mathrm{max}|e|$: maximum absolute error ($\max_i\left|s_i-r_i \right|$); RMSE: root mean square error ($\sqrt{\frac{1}{n}\sum_{i = 1}^n\left(s_i-r_i\right)^2}$); MARE: mean absolute relative error ($\frac{1}{n}\sum_{i = 1}^n\left|\frac{s_i-r_i}{r_i}\right|\times 100$), where $n$ denotes the total number of frames, $i$ denotes index of each frame, $s_i$ denotes the SDM-predicted MLD at frame $i$, and $r_i$ denotes the FEM-predicted MLD at frame $i$.

## 4. Discussion

In these two proof-of-concept cases, the SDM showed several practical advantages for computational stent simulation. A key feature of the SDM is that its approximation, derived from Newton’s laws of motion, retains physical entities that are updated at each time step. Unlike FEM, this approach eliminates the need for computing large matrices, resulting in greater simplicity and ease of use. Additionally, polygonal meshes are utilized to compute normal vectors, allowing for an efficient representation of the artery’s complex geometry.

Benchmark tests revealed several key advantages of the SDM for computational stent simulation. First, it provided consistent results regardless of the number of vertices, enabling accurate simulations without requiring an excessively fine mesh. This significantly reduces the burden of mesh discretization and computational time. Additionally, the framework effectively captured the mechanical behavior of both the stent and artery through the successful implementation of material properties. With its simplified formulation and efficient handling of complex geometries, the SDM offers a computationally efficient yet accurate alternative to traditional methods.

Unlike previous studies that have predominantly focused on self-expandable stents, as shown in table 7, the present study demonstrates the application of the SDM to balloon-expandable stents. This highlights the adaptability of the SDM in modeling different stent types while maintaining computational efficiency. The SDM has primarily been applied to self-expandable stents because it effectively models internal forces through tangential and normal components, capturing their inherent flexibility. However, this model lacks a detailed representation of the mechanical behavior of both the stent and the artery, simplifying their representation without fully capturing the complexity of their mechanical interactions. A notable study by Djukic *et al* (2019) applied the SDM to coronary artery simulations. Although the specific stent type was not specified, it is reasonable to assume that a balloon-expandable stent was used, as these are commonly employed in coronary interventions. FEM was incorporated in their study to compute external forces based on the assumptions of the SDM. While the inclusion of FEM may enhance accuracy, it also increases computational time. In contrast, the present model does not require additional computational interventions while still effectively capturing the mechanical behaviors of both the stent and the artery, thereby offering a balanced estimation of reliability and simplicity.

**Table 7.** Literature review of comparative studies on the simplex deformable model.

Study	Application	Stent type	Computational platform	Time (s)
Flórez Valencia *et al* (2007)	Aorta	Self-expandable	Intel® Pentium III 800 MHz	1.80
Larrabide *et al* (2012)	Intracranial artery	Self-expandable	Intel® Core$ ^{\mathrm{TM}}$ Duo T7300 2.0 GHz	7–59
McFarlane *et al* (2013)	Peripheral artery	Self-expandable	Intel® Core$ ^{\mathrm{TM}}$ i5	0.5–3
Paliwal *et al* (2016)	Internal carotid artery, basilar artery	Self-expandable	N/A	N/A
Chen *et al* (2018)	Thoracic aortic	Self-expandable	Intel® Core$ ^{\mathrm{TM}}$ i7-6700 K 4.00 GHz	105
Djukic *et al* (2019)	Coronary artery	Balloon-expandable	CPU + GPU$^*$	N/A
Lee *et al* (2026, current)	Coronary artery	Balloon-expandable	Intel® Core$ ^{\mathrm{TM}}$ i9-10 900X 3.70 GHz	10–22

*Note:* N/A indicates data were not available in the cited source. ${}^*$A graphics processing unit (GPU) was integrated.

However, the current study has several limitations. First, the stent was assumed to be purely elastic, which excludes the consideration of permanent plastic deformation on stent during the expansion process. Second, the stent in this framework was uniformly expanded along its length, whereas in FEM simulations using Abaqus, as well as in real clinical procedures, balloon expansion undergoes straightening. This simplified expansion mechanism in the SDM may contribute to the local discrepancies observed between the SDM and FEM results. Thirdly, the balloon deflation phase and the resulted stent-artery recoil were not considered. Moreover, while this study did not provide a detailed representation of the stent’s material and mechanical behavior, the results obtained using the simplex deformable mesh showed good agreement with those generated by FEM, as demonstrated in the Results section. Lastly, although a hyperelastic material property-specifically, the 6th-order reduced polynomial model was employed to accurately represent the homogeneous properties of normal artery tissue, this study did not account for material heterogeneity, such as regions with calcification, fibrosis, or lipid-rich plaque.

## 5. Conclusions

This study presents a proof-of-concept implementation of the SDM for computational simulations of balloon-expandable stenting in two patient-specific 3D coronary anatomies. In these two patient-specific cases, the SDM provided good compatibility with FEM while offering faster simulation and fewer vertices. Unlike existing studies on virtual stenting simulation using the SDM, the present study advances the SDM for balloon-expandable coronary stents by embedding stent material and structural parameters directly into the internal force term, enabling mechanics-informed deployment prediction without resorting to full FEM-based force evaluation. Future work will focus on capturing the precise mechanical behavior of the stent and balloon, including the recoil effect resulting from the elastoplastic material properties of the stent and the straightening of the balloon during expansion. Additionally, the material characteristics of the artery, including calcium, fibrosis, and lipid, will be incorporated into the simulations.

## Data Availability

The data cannot be made publicly available upon publication because they contain sensitive personal information. The data that support the findings of this study are available upon reasonable request from the authors.

## Appendix A Discrete SDM motion equations

Let assume that time is discretized as $t_i = t_0 + \mathrm{i}\Delta t$ and then equation (3) is integrated over time using finite difference method. Since $P_i^t$ is the position of vertex $i$ at time $t$, the velocity and acceleration approximation using Euler’s method with the centered difference method are given as:

\begin{equation*} \begin{split} \dfrac{\partial P_i}{\partial t} &amp;\approx \dfrac{P_i^{t+\Delta t} - P_i^{t-\Delta t}}{2\Delta t} \\ \dfrac{\partial^2 P_i}{\partial t^2} &amp;\approx \dfrac{P_i^{t+\Delta t} - 2P_i^t - P_i^{t-\Delta t}}{\Delta t^2}. \end{split}\end{equation*}

Substituting equation (9), equation (3) is defined as:

\begin{equation*} \begin{split} m\dfrac{P_i^{t+\Delta t} - 2P_i^t - P_i^{t-\Delta t}}{\Delta t^2} &amp; = -\gamma\dfrac{P_i^{t+\Delta t} - P_i^{t-\Delta t}}{2\Delta t}+ F^t_\mathrm{int} + F^t_\mathrm{ext}\\ P_i^{t+\Delta t} - 2P_i^t - P_i^{t-\Delta t} &amp; = \dfrac{\Delta t^2}{m}\left(-\gamma\dfrac{P_i^{t+\Delta t} - P_i^{t-\Delta t}}{2\Delta t}+ F^t_\mathrm{int} + F^t_\mathrm{ext}\right)\\ P_i^{t+\Delta t} - 2P_i^t - P_i^{t-\Delta t} &amp; = -\dfrac{\gamma\Delta t}{m}\left(P_i^{t+\Delta t} - P_i^{t-\Delta t}\right) + \dfrac{\Delta t^2}{m}\left(F^t_\mathrm{int} + F^t_\mathrm{ext}\right). \end{split}\end{equation*}

The damping term is approximated based on the centered difference method as:

\begin{equation*} P_i^{t+\Delta t} - P_i^{t-\Delta t} \approx 2\left(P_i^t - P_i^{t-1}\right).\end{equation*}

Therefore, equation (11) can be rewritten as:

\begin{equation*} P_i^{t+\Delta t} = \left(2-\dfrac{\gamma\Delta t}{m}\right)P_i^t - P_i^{t-\Delta t} + \dfrac{\gamma\Delta t}{m}P_i^{t-1} + \dfrac{\Delta t^2}{m}\left(F^t_\mathrm{int} + F^t_\mathrm{ext}\right).\end{equation*}

Assuming if the time step is normalized, $\Delta t^2/m$ is absorbed into force terms and if the time step $\Delta t$ is small relative to the characteristic timescale of the system, it can be assumed as $\gamma\Delta t/m \approx \gamma$. That is $m$ is absorbed into the definition of $\gamma$, treating it as a dimensionless damping coefficient. Thus, equation (12) can be simplified as follows:

\begin{equation*} P_i^{t+\Delta t} = \left(2-\gamma\right)P_i^t - P_i^{t-\Delta t} + \gamma P_i^{t-1} + \left(F_\mathrm{int}^t + F_\mathrm{ext}^t\right).\end{equation*}

Using a discrete time index, equation (13) can be rewritten as:

\begin{equation*} P_i^{t+1} = P_i^t + \left(1-\gamma\right)\left(P_i^t - P_i^{t-1}\right) + F_\mathrm{int}^t + F_\mathrm{ext}^t\end{equation*}

where rewriting $P_i^{t+\Delta t}$ as $P_i^{t+1}$ and $P_i^{t-\Delta t}$ as $P_i^{t-1}$ is a common notation convention for discrete time steps, respectively.

## Appendix B Constitutive model

The strain energy density function for the reduced polynomial hyperelastic model is given in Abaqus as:

\begin{equation*} W = \sum_{i = 1}^N {C_{i0}\left(\bar{I}_1-3\right)^i}\end{equation*}

where $\bar{I}_1 = J^{-1/3}I_1$ denotes the modified isochoric first invariant of the deformation tensor, and $J = \mathrm{det}F$ is the Jacobian of the deformation gradient. The absence of an explicit volumetric energy term implies an incompressible material behavior. However, since no additional constraint (such as a Lagrange multiplier to enforce $\mathrm{det}F = 1$) is included, the model behaves advances as nearly-incompressible and may allow small volume changes in practice. The Cauchy stress $\sigma$ is obtained by differentiating the strain energy density function with respect to the principal stretch $\lambda$:

\begin{equation*} \sigma = \dfrac{\partial W}{\partial \lambda} = \left(2\lambda - 2\lambda^{-2}\right)\sum_{i = 1}^N {C_{i0}i\left(\bar{I}_1-3\right)^{i-1}}\end{equation*}

where the modified first invariant simplifies to $\bar{I}_1 = \lambda^2 + 2\lambda^{-1}$. In this study, a sixth-order reduced polynomial model was employed, resulting in the following explicit form of equation (16):

\begin{align*} \sigma &amp; = \left(2\lambda - 2\lambda^{-2}\right)\left[C_{10} + 2C_{20}\left(\bar{I}_1-3\right) + 3C_{30}\left(\bar{I}_1-3\right)^2\right.\nonumber\\ &amp; \quad + \left. 4C_{40}\left(\bar{I}_1-3\right)^3 + 5C_{50}\left(\bar{I}_1-3\right)^4 + 6C_{60}\left(\bar{I}_1-3\right)^5\right]\end{align*}
